# Predictors of Functional Recovery among Musculoskeletal Oncology Patients Undergoing Lower Extremity Endoprosthetic Reconstruction

**DOI:** 10.3390/curroncol29100600

**Published:** 2022-10-13

**Authors:** Aaron M. Gazendam, Patricia Schneider, Diane Heels-Ansdell, Mohit Bhandari, Jason W. Busse, Michelle Ghert

**Affiliations:** 1 Division of Orthopaedic Surgery, McMaster University, Hamilton, ON L8S 4L8, Canada; 2 Department of Health Research Methods, Evidence, and Impact, McMaster University, Hamilton, ON L8S 4L8, Canada; 3 The Michael G. DeGroote Institute for Pain Research and Care, McMaster University, Hamilton, ON L8S 4L8, Canada; 4 Department of Anesthesia, McMaster University, Hamilton, ON L8S 4L8, Canada; 5 The Chronic Pain Centre of Excellence for Canadian Veterans, Hamilton, ON L8P 0A1, Canada

**Keywords:** Toronto extremity salvage score, functional outcomes, orthopaedic oncology, sarcoma

## Abstract

Background and Objectives: Functional outcomes are important for oncology patients undergoing lower extremity reconstruction. The objective of the current study was to describe patient reported function after surgery and identify predictors of postoperative function in musculoskeletal oncology patients undergoing lower extremity endoprosthetic reconstruction. Methods: We performed a cohort study with functional outcome data from the recently completed *Prophylactic Antibiotic Regimens in Tumor Surgery* (PARITY) trial. We utilized the 100-point Toronto Extremity Salvage Score (TESS), which was administered pre-operatively and at 3, 6 and 12 months post-operatively. Higher scores indicate better physical functioning, and the minimally important difference is 11 points. We calculated mean functional scores at each timepoint after surgery and developed a logistic regression model to explore predictors of failure to achieve excellent post-operative function (TESS ≥ 80) at 1 year after surgery. Results: The 555 patients included in our cohort showed important functional improvement from pre-surgery to 1 year post-surgery (mean difference 14.9 points, 95%CI 12.2 to 17.6; *p* < 0.001) and 64% achieved excellent post-operative function. Our adjusted regression model found that poor (TESS 0–39) pre-operative function (odds ratio [OR] 3.3, 95%CI 1.6 to 6.6); absolute risk [AR] 24%, 95%CI 8% to 41.2%), older age (OR per 10-year increase from age 12, 1.32, 95%CI 1.17, 1.49; AR 4.5%, 95%CI 2.4% to 6.6%), and patients undergoing reconstruction for soft-tissue sarcomas (OR 2.3, 95%CI 1.03 to 5.01; AR 15.3%, 95%CI 0.4% to 34.4%), were associated with higher odds of failing to achieve an excellent functional outcome at 1-year follow-up. Patients undergoing reconstruction for giant cell tumors were more likely to achieve an excellent functional outcome post-operatively (OR 0.40, 95%CI 0.17 to 0.95; AR −9.9%, 95%CI −14.4% to −0.7%). Conclusions: The majority of patients with tumors of the lower extremity undergoing endoprosthetic reconstruction achieved excellent function at 1 year after surgery. Older age, poor pre-operative function, and endoprosthetic reconstruction for soft tissue sarcomas were associated with worse outcomes; reconstruction for giant cell tumors was associated with better post-operative function. Level of Evidence: Therapeutic Level IV.

## 1. Introduction

Surgical intervention with wide excision and negative margins is the mainstay of treatment for patients with malignant bone tumors [1,2]. With improvements in imaging modalities, chemotherapeutic agents and surgical techniques, limb-salvage surgery has become the standard of care for the majority of patients diagnosed with malignant bone tumors of the extremity [3]. Limb-salvage surgery allows for the same oncologic control as amputations with potential improvements in function and quality of life [4].

In skeletally mature patients with tumors centred on the hip and knee, endoprosthetic reconstruction has become the reconstruction technique of choice [5]. Endoprostheses are intended to reproduce the form and function of the limb following large bony resections. Originally designed for primary bone tumors, the indications have been extended to patients with metastatic bone disease, fractures and revision arthroplasty.

There has been an increased interest in functional outcomes following limb-salvage surgery [6,7,8,9]; however, there remain important knowledge gaps. The majority of the literature consists of small, single center, retrospective reviews with the inherent biases such study designs carry [10]. Further, functional outcomes are most commonly reported at a single time point post-operatively, which does not allow for an assessment of change over time [11,12,13]. Due to the invasive nature of oncologic resections and reconstructions, some patients may experience prolonged functional impairment after surgery. This information is critical to inform patients and clinicians about the expected rehabilitation course following endoprosthetic reconstruction [14].

This study aimed to: (1) describe changes in patient-reported functional outcomes pre-operatively to 1 year post-operatively following lower extremity endoprosthetic reconstruction, and (2) identify pre-operative patient and tumor variables associated with post-operative function.

## 2. Methods

### 2.1. Study Design and Setting

This was a secondary analysis of the recently completed PARITY (Prophylactic Antibiotic Regimens in Tumor Surgery) trial [15]. PARITY was a multicenter randomized controlled trial (RCT), in which surgeons, patients and outcome assessors were blinded, that investigated the impact of 24 h vs. 5 days of post-operative intravenous prophylactic antibiotics on surgical site infections among patients undergoing endoprosthetic reconstruction of lower extremity bone and soft-tissue tumors. Eligible patients included individuals aged 12 years or older with a primary bone tumor of soft tissue sarcoma invading the femur or tibia or patients with oligometastatic bone disease of the femur or tibia with expected survival of at least 1 year with a planned endoprosthetic reconstruction. Patients were excluded if they had previous surgical site infections at the surgical site or were known to be colonized with methicillin-resistant *Staphylococcus aureus* or vancomycin resistant *Enterococcus.* This trial was registered [NCT01479283] and received ethics approval from the Hamilton Integrated Research Ethics Board (REB# 12-009). The PARITY trial consisted of 48 clinical sites in Canada, the United States, Argentina, Australia, Austria, Brazil, Egypt, India, the Netherlands, Singapore, South Africa and Spain. Patients were followed until 1 year post-operatively and assessed by their treating surgeon at 3, 6 and 12 months post-operatively. The current study followed the Strengthening the Reporting of Observational Studies in Epidemiology (STROBE) guidelines for reporting of observational studies [16].

### 2.2. Participants

All patients who underwent a proximal femur reconstruction (PFR), distal femur reconstruction (DFR) or proximal tibia reconstruction (PTR) with an endoprosthesis as part of the PARITY trial were included in the current analysis.

### 2.3. Data Sources and Variables

Baseline patient demographic, tumor characteristics, surgical data and functional outcome scores were obtained from the prospectively collected PARITY trial database.

### 2.4. Functional Outcomes

The Toronto Extremity Salvage Score (TESS) was designed to address the World Health Organization’s definitions of Disability, Impairment, and Handicap [17]. It is a 30-item, patient-reported questionnaire that focuses on the ability to perform activities of daily living in a variety of daily settings. Scores are translated to a 0–100 scale with higher scores representing better function. In the PARITY trial, the TESS was administered pre-operatively, and at 3, 6 and 12 months post-operatively. We categorized TESS values as poor (0–39), fair (40–59), good (60–79) or excellent (80–100). A TESS of ≥80 is commonly reported by unoperated healthy controls aged 30–69 [18], and the minimally important difference (MID) is 11 points [19].

### 2.5. Statistical Analysis

Demographic data were reported using descriptive statistics, with mean and standard deviation (SD) or median and interquartile range, depending on data distribution. Patient-reported functional outcomes were presented as means and SDs at all time points (pre-operative, 3 months, 6 months and 12 months), both for the entire cohort and stratified by PFRs, DFRs and PTRs. Change scores were presented as mean differences (MD) and 95% confidence intervals (CI). We explored for statistical significance of functional changes pre-operatively to each post-operative follow-up using paired t-tests.

We constructed a multivariable logistic regression model to explore predictors of failure to achieve excellent functional outcome (TESS ≥ 80) at 1-year follow-up. We pooled patients into a single group for the current study as the PARITY trial was a no-difference study. All patients with complete data were included in our regression analysis. We selected six covariates previously reported as predictors or judged by our clinical experts to be related to functional outcomes: age, gender, tumor type (primary bone sarcoma, STS invading bone, metastatic bone disease, or giant cell tumor [GCT]), endoprosthetic reconstruction (PFR, DFR or PTR), systematic metastases at presentation, and pre-operative TESS [20,21,22]. We also adjusted for antibiotic treatment (24 h vs. 5 day). We excluded independent variables with fewer than 40 observations, unless we were able to collapse them with other related variables to exceed this threshold, to provide reassurance that each variable had sufficient discriminant power to detect an association with functional outcome if such an association existed. To avoid over-fitting, we required at least 10 events and 10 non-events per category of independent variable, for a minimum of 120 patients who achieved an excellent functional outcome, and 120 that did not by 1 year after surgery [23]. Model performance was evaluated using the Hosmer–Lemeshow statistic to assess for goodness-of-fit [24]. Outcomes of the binomial logistic regression were presented with odds ratios (OR) and 95% confidence intervals (CI). We calculated the absolute risk (AR) for each significant predictor and estimated the baseline risk for failure to achieve an excellent function outcome at 1 year by calculating the incidence among patients without any significant risk factors. All analyses were performed using SPSS (IBM SPSS Statistics for Mac, V26). A value of *p* < 0.05 was considered to be significant for all analyses.

## 3. Results

### 3.1. Cohort Characteristics

There were 895 patients who were screened for eligibility in the PARITY trial with 604 meeting the eligibility requirements, enrolling and included in the final analysis. Of the 604 patients enrolled in the PARITY trial, 555 underwent endoprosthetic reconstruction of the proximal femur (n = 144), distal femur (n = 312) or proximal tibia (n = 99), and had patient-reported functional outcome data available for one or more post-operative time points. Of the 49 excluded patients, 15 had a non-eligible endoprosthetic reconstruction and 34 were missing TESS data at all follow-ups. The mean age of the cohort was 41 (SD ± 22) and 60% were male (332/555). The most common diagnosis was a primary bone sarcoma (n = 407) followed by an STS (n = 54), metastatic bone disease (n = 51), and a GCT (n = 43). The mean follow-up was 333 days (range 2–366) with 51 (9%) patients dying from disease progression prior to the final follow-up (Table 1).

Differences among subgroups were found (Table 1). Patients undergoing PFR were older, more likely to present with a metastatic bone lesion with systemic metastases and die of disease progression. Differences in pre-operative TESS scores were found between groups.

### 3.2. Functional Outcomes

**Figure 1:**
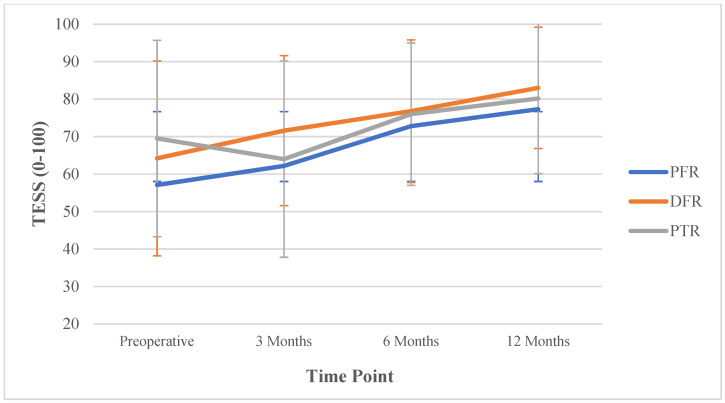
Changes in the TESS scores over time with points indicating means and error bars indicating standard deviations. PFR: proximal femur reconstruction, DFR: distal femur reconstruction, PTR: proximal tibia reconstruction.

Mean functional outcome scores increased over time for the cohort, and the average TESS at the 12-month follow-up was 81.1 (SD ± 17.8) (Table 2). There were statistically significant improvements in the TESS from pre-operative to final follow-up that exceeded the MID of 11 (MD 14.9 [95%CI; 12.2, 17.6] *p* < 0.001) (Table 3, Figure 1). There were differences in improvement in pre-operative to 12-month TESS based on anatomic location, with PFR (MD 16.6 [95%CI; 10.6, 22.6], *p* < 0.001) and DFR (MD 16.5 [95%CI; 13.0, 20.0], *p* < 0.001) showing larger improvements than PTR (MD 8.2 [95%CI; 1.8, 14.6], *p* = 0.013).

### 3.3. Binomial Logistic Regression Analysis

We included 397 of the patients with both pre-operative and 12-month follow-up TESS in our regression analysis. There were 51 patients who died prior to the one-year follow-up and 107 with missing TESS data at their pre-operative or 12-month follow-up. Of the 397 patients, 254 (64%) achieved an excellent (TESS ≥ 80) outcome. Our adjusted regression model found that poor (TESS < 40) pre-operative function (OR 3.3, 95%CI 1.64, 6.60; AR 24%, 95%CI 8.0, 41.2), older age (OR per 10-year increase from age 12, 1.32, 95%CI 1.17, 1.49; AR 4.5% per decade, 95%CI 2.4, 6.6), and STSs (OR 2.27; 95%CI 1.03, 5.01; AR 15.3%, 95%CI 0.4, 34.4) were less likely to achieve an excellent functional outcome; patients presenting with GCTs were more likely to achieve an excellent functional outcome (OR 0.40, 95%CI 0.17, 0.95; AR −9.9%, 95%CI −14.4, −0.7). Patient sex, metastases at presentation, type of endoprosthetic reconstruction (PFR, DFR or PTR) and antibiotic duration group were not associated with excellent patient-reported function 1 year after surgery. (Table 4) Our model demonstrated goodness of fit according to the Hosmer– Lemeshow test (χ^2^ = 9.03, *p* = 0.340).

## 4. Discussion

We found that patients with bone tumors undergoing endoprosthetic reconstruction of the lower extremity demonstrate important functional improvement at 1-year follow-up, with approximately two-thirds achieving excellent functioning. Older patients, reporting poor pre-operative functioning, and presenting with an STS, were less likely to report excellent function at 1 year; patients presenting with a GCT were more likely to achieve excellent long-term functional recovery.

The strengths of our study include a large, comprehensive analysis of prospectively collected functional scores in patients undergoing lower extremity endoprosthetic reconstruction. Second, we recruited patients from 48 clinical sites in 12 countries which increases the generalizability of our findings. Third, this is the first study to capture changes from pre-operative function over the course of a patient’s rehabilitation in this population. Fourth, we had very little missing data (6%) in our cohort. Our study does have some limitations. We captured functional outcomes up to 1 year after surgery and it remains possible that additional recovery may have been seen after this time, particularly among the 25% of patients that required re-operation. Secondly, the results of the current study should not be extrapolated to patients undergoing endoprosthetic reconstruction for non-oncologic indications such as fracture or revision surgery.

Given the surgically complex nature of tumor resections and reconstructions, there may be concerns that patients are left with significant functional limitations [25]; however, the current study demonstrates that most musculoskeletal oncology patients achieve excellent long-term function at 1 year post-operatively. Compared to other types of reconstruction, patients undergoing PTRs showed a decrease in function at 3 months after surgery. The majority of patients undergoing PTRs require extensor mechanism reconstruction, typically through a gastrocnemius rotation flap with a wire or suture fixation to reconstruct the patellar tendon [26,27]. The post-operative rehabilitation protocol for these patients generally involves a prolonged period of immobilization which was likely a contributing factor in the significant reduction in function noted at the 3-month follow-up visit [27].

Patients with poor pre-operative functional scores (TESS < 40) were at a higher risk of not achieving optimal post-operative functional status, independent of age, tumor type or anatomic location. Similar findings have been demonstrated in other orthopaedic populations, including patients undergoing total hip arthroplasty [28].

Patients with an STS requiring bone resection and endoprosthetic reconstruction were significantly less likely to achieve optimal function in this cohort. Bony invasion in the STS is relatively rare and often indicates a larger, more aggressive tumor [21]. Soft-tissue sarcomas often necessitate more soft tissue and muscle resection than primary bone tumors to achieve negative margins, which has the potential to negatively impact post-operative function. High-grade STSs are also generally managed with peri-operative radiotherapy. Given the risk of wound healing and periprosthetic infection associated with pre-operative radiation, some clinicians opt for post-operative radiation [29,30]. However, post-operative radiation is associated with soft tissue fibrosis, stiffness and lymphedema which may negatively impact functional outcomes [30].

Older age was a risk factor for failure to achieve excellent post-operative function, and this has been shown in previous studies of primary bone tumor resection and endoprosthetic reconstruction [31] and lower extremity limb salvage surgery [20]. With increasing age, patients are more likely to present with frailty; representing an age-related decline in function, sarcopenia and energy which impacts their ability to recover post-operatively [32]. Similarly, older patients are more likely to have medical comorbidities which may impact their ability to rehabilitate. Further research evaluating the utility of prehabilitation to optimize post-operative functional recovery is warranted, perhaps particularly in older patients and those with lower pre-surgical functioning [33].

Identifying predictors of post-operative function regain following lower extremity endoprosthetic will allow both patients and physicians to make evidenced-based decisions when discussing alternative management strategies. Additionally, recognizing patients at the highest risk of failure allows clinicians to appropriately allocate resources to ensure high-risk patients are given the best chance of success. For example, high-risk patients may benefit from additional pre- and post-operative rehabilitation to ensure their post-operative function is optimized.

## 5. Conclusions

Most patients with tumors of the lower extremity undergoing endoprosthetic reconstruction achieved excellent function at 1 year after surgery. Older age, poor pre-operative function, and endoprosthetic reconstruction for soft tissue sarcomas were associated with worse outcomes; reconstruction for giant cell tumors were associated with better post-operative function.

## Figures and Tables

**Table 1:** Patient characteristics.

Variable	Entire Cohort (n = 555)	Proximal Femur Reconstruction (n = 144)	Distal Femur Reconstruction (n = 312)	Proximal Tibia Reconstruction (n = 99)	Subgroup Differences (*p*-Value)
Age (SD)	40.7 (21.6)	51.3 (20.4)	37.1 (20.9)	36.6 (20.6)	<0.001 **
Gender (M/F)	332/223	91/53	177/135		0.237 *
Diagnosis (%)					
Primary bone sarcoma	407 (73)	97 (67)	239 (77)	71 (71)	<0.001 *
Soft tissue sarcoma	54 (10)	11 (8)	33 (11)	10 (10)	
Metastatic bone disease	51 (9)	32 (22)	16 (5)	3 (3)	
Giant cell tumor	43 (8)	4 (3)	24 (7)	15 (15)	
Pre-operative TESS (%)					
Poor (0–39)	113 (20)	44 (31)	58 (19)	11 (11)	0.003 *
Fair (40–59)	96 (17)	22 (15)	53 (17)	21 (21)	
Good (60–79)	140 (25)	23 (16)	96 (31)	21 (21)	
Excellent (80–100) Missing	180 (32) 26 (5)	45 (31) 10 (7)	92 (29) 13 (4)	43 (43) 3 (3)	
Systemic Metastases (%)					
Yes	97 (17)	42 (29)	45 (14)	10 (10)	<0.001 *
No	458 (83)	102 (71)	267 (86)	89 (90)	
Death from disease progression (%)	51 (9)	23 (16)	24 (8)	4 (4)	0.003 *

SD = standard deviation; M = male; F = female; TESS = Toronto Extremity Salvage Score; * Chi-squared; ** ANOVA (analysis of variance).

**Table 2:** Functional outcome scores over time.

Functional Score	Overall Mean Score (SD)	PFR Mean Score (SD)	DFR Mean Score (SD)	PTR Mean Score (SD)
TESS				
Preoperative	63.5 (27.7)	57.1 (31.2)	64.2 (26.0)	69.5 (26.2)
3 months	67.9 (21.3)	62.2 (21.7)	71.6 (20.0)	64.0 (26.2)
6 months	75.7 (19.2)	72.8 (19.3)	76.8 (19.0)	76.0 (19.0)
12 months	81.1 (17.8)	77.3 (18.5)	83.0 (16.2)	80.2 (20.1)

TESS = Toronto Extremity Salvage Score; SD = standard deviation; PFR = proximal femur reconstruction; DFR = distal femur reconstruction; PTR = proximal tibia reconstruction.

**Table 3:** TESS change scores over time.

Functional Score	Mean Differences (95%CIs)					
	0–3 Months	*p*-Value	0–6 Months	*p*-Value	0–12 Months	*p*-Value
TESS						
Overall	3.4 (0.7, 6.2)	0.015	10.0 (7.4, 12.6)	<0.001	14.9 * (12.2, 17.6)	**<0.001**
PFR	2.7 (−3.7, 9.0)	0.410	12.3 * (6.1, 18.6)	<0.001	16.6 * (10.6, 22.6)	**<0.001**
DFR	7.1 (3.8, 10.5)	**<0.001**	10.8 (7.5, 14.0)	<0.001	16.5 * (13.0, 20.0)	**<0.001**
PTR	−7.0 (−13, −0.5)	0.034	4.8 (−1.1, 10.7)	0.11	8.2 (1.8, 14.6)	0.013

TESS = Toronto Extremity Salvage Score; CI = confidence interval; PFR = proximal femur reconstruction; DFR = distal femur reconstruction; PTR = proximal tibia reconstruction; **bolded** = statistically significant when evaluated with paired t-tests; * exceeds minimal important difference cut-off.

**Table 4:** Binomial logistic regression analysis evaluating factors associated with failure to achieve excellent post-operative function at 1 year (n = 397).

Factor	OR (95%CI)	*p*-Value	Absolute Risk, % (95%CI) *
Age (per 10-year increase from age 12)	1.32 (1.17, 1.49)	<0.001	4.5 (2.4, 6.6)
Sex Female Male	reference category 1.00 (0.63, 1.60)	0.999	
Tumor Type Bone sarcoma Soft-tissue sarcoma Metastatic bone disease Giant cell tumor	reference category 2.27 (1.03, 5.01) 0.78 (0.28, 2.20) 0.40 (0.17, 0.95)	0.042 0.628 0.038	15.3 (0.4, 34.4) −9.9 (−14.4, −0.7)
Type of Reconstruction Distal femur Proximal femur Proximal tibia	reference category 0.98 (0.55, 1.75) 1.3 (0.72, 2.4))	0.947 0.368	
Pre-operative TESS Score Excellent (80–100) Good (60–79) Fair (40–59) Poor (0–39)	reference category 1.04 (0.57, 1.91) 1.83 (0.96, 3.50) 3.30 (1.6, 6.60)	0.889 0.068 0.001	24.0 (8.0, 41.2)
Metastases at Presentation	1.30 (0.61, 2.62)	0.537	
Antibiotic Duration 24 h regime 5-day regime	Reference 0.91 (0.58, 1.42)	0.668	

OR = odds ratio, CI = confidence interval, * absolute risks are reported for significant factors in the adjusted model.

## Data Availability

Data to be made available upon request.
